# Nickel–Titanium Rotary Instruments: Mechanical and Metallurgical Characteristics

**DOI:** 10.3390/clinpract12010012

**Published:** 2022-02-14

**Authors:** Alessio Zanza, Rodolfo Reda, Luca Testarelli

**Affiliations:** Department of Oral and Maxillofacial Sciences, Sapienza University of Rome, 00161 Rome, Italy; alessio.zanza@uniroma1.it (A.Z.); luca.testarelli@uniroma1.it (L.T.)

During the last two decades, the field of the nickel–titanium (NiTi) rotary instruments has been thoroughly changed by the latest, exciting innovations in both manufacturing technologies and the knowledge of their mechanical performance. Since the 1990s, the world of endodontics has made considerable progress due to the great number of studies in recent years, leading to a deeper understanding of the mechanical and metallurgical behaviour of NiTi instruments [1,2]. The improvements in endodontic instruments are a result of a fundamental focus on two aspects, that aim to reduce their intracanal fracture as much as possible: the instruments’ design and geometry and the alloy with its corresponding heat treatments [3,4]. In fact, the most remarkable limitation of NiTi instrumentation is the possibility of instrument failure during shaping procedures. As thoroughly demonstrated, the two main causes of this are undoubtedly the cyclic fatigue and the excessive torsional loads exerted on instruments during the dentinal cutting in curved canals. With the purpose of reducing failures and increasing the speed and the predictability of root canal treatments, researchers and engineers have focused their attention on the factors related to the geometry and design of the instruments that are able to influence their mechanical resistance, both in terms of torsional and cyclic fatigue [5].

Regarding cyclic fatigue resistance, Grande et al. demonstrated that mass plays a key role: the lower the mass, the greater the cyclic fatigue resistance. Moreover, there are several parameters that influence mass such as cross-sectional shape, diameter, core mass, flute depth, and the number of spirals and tapers, yet all of these parameters have a direct correlation with the total mass of the instrument at the point of maximum curvature, where the flexural moments are higher. For this reason, it is reasonable to consider mass as the most significant factor [6,7].

On the other hand, the relationship between mass and torsional resistance appears to be more complicated. As stated by Zanza et al. the mass and, consequently, the volume of instruments are not crucial in terms of absolute value; however, they are highly significant parameters when they are spread in relation to the rotational centre. This geometrical aspect can be translated using the concept of polar moment of inertia, which considers the distribution of volume in relation to the pivot centre [8,9].

However, in order to assess the mechanical performance of NiTi instruments and their effectiveness during root canal treatments, static tests that aim to separately evaluate cyclic fatigue and torsional resistance are insufficient [10,11]. In fact, in order to obtain more reliable results, the reciprocal correlation between flexural and torsional stresses should be considered since, during shaping procedures, instruments are constantly subjected to a combination of these stresses [12,13]. Thus, the determination of the dynamic behaviour of instruments is of vital importance [14]. For this reason, the evaluation of the cutting efficiency and the shaping ability of NiTi instruments should be performed during the assessment of their mechanical properties. It is not uncommon to have instruments with a high cyclic fatigue and torsional resistance, requiring a high apical pressure to reach a working length. This could be directly related to the intrinsic properties of the alloy, such as the hardness of the surface or the crystallographic phase, and to the geometry and design of the instruments [4,15].

According to this idea, the geometry and design are not necessarily the unique aspects of rotary instruments that could influence their performance. As demonstrated, the alloy composition, as a percentage of nickel and titanium, and its crystallographic phase are fundamental as they have a significant impact on the mechanical performance of NiTi instruments [16]. The NiTi alloy can be organized into two different crystallographic forms depending on the applied stress and temperature changes, the austenite and the martensite. The first alloy is characterized by an increased superelasticity; it is stiffer and more stable at higher temperatures than martensite. On the contrary, the second alloy is more flexible, ductile and shows an increased resistance to cyclic fatigue. According to this, the conventional NiTi alloy is stable in the austenitic phase at an ambient and intracanal temperature; however, in recent decades, manufacturers have developed heat treatments that allow instruments to assume a martensitic form even at ambient or intracanal temperatures [3]. As a consequence of the different mechanical behaviours of NiTi alloys depending on temperature, it is fundamental to perform in vitro static and dynamic tests at simulated body temperature in order to obtain clinically significant results. Tests that are conducted above body temperature (35.1 °C ± 1 °C) should be regarded as ineffectual, and thus should not be considered as representative of the effectiveness of NiTi alloys [4,17].

In conclusion, the evaluation and comparison of the performance of endodontic instruments should be assessed trough a multimethod approach comprising the assessment of both static and dynamic mechanical behaviour that considers the reciprocal influence of different stresses and metallurgical properties, since these two aspects are strictly related each other and should not be evaluated separately; this approach could lead to misleading or incomplete conclusions [18]. Moreover, considering the lack of standardization regarding the testing models used for the comparison and determination of the physical properties of NiTi instruments, further research and reliable, standardized methodologies are required for an in-depth evaluation of fracture properties and the impact of one specific parameter and its relationship with other parameters on the characteristics of NiTi instruments.
